# Potential pathophysiological role of the ion channel TRPM3 in myalgic encephalomyelitis/chronic fatigue syndrome (ME/CFS) and the therapeutic effect of low-dose naltrexone

**DOI:** 10.1186/s12967-024-05412-3

**Published:** 2024-07-05

**Authors:** Matthias Löhn, Klaus Josef Wirth

**Affiliations:** 1 Institute for General Pharmacology and Toxicology, University Hospital, Goethe University, Frankfurt am Main, Germany; 2 Mitodicure GmbH, D-65830 Kriftel, Germany

**Keywords:** TRPM3 channel, Myalgic Encephalomyelitis/Chronic fatigue syndrome, ME/CFS, Post-COVID syndrome, Long-COVID, Exercise intolerance, GABA, Small fiber neuropathy, Naltrexone

## Abstract

Myalgic Encephalomyelitis/Chronic Fatigue Syndrome (ME/CFS) is a debilitating disease with a broad overlap of symptomatology with Post-COVID Syndrome (PCS). Despite the severity of symptoms and various neurological, cardiovascular, microvascular, and skeletal muscular findings, no biomarkers have been identified. The Transient receptor potential melastatin 3 (TRPM3) channel, involved in pain transduction, thermosensation, transmitter and neuropeptide release, mechanoregulation, vasorelaxation, and immune defense, shows altered function in ME/CFS. Dysfunction of TRPM3 in natural killer (NK) cells, characterized by reduced calcium flux, has been observed in ME/CFS and PCS patients, suggesting a role in ineffective pathogen clearance and potential virus persistence and autoimmunity development. TRPM3 dysfunction in NK cells can be improved by naltrexone in vitro and ex vivo, which may explain the moderate clinical efficacy of low-dose naltrexone (LDN) treatment. We propose that TRPM3 dysfunction may have a broader involvement in ME/CFS pathophysiology, affecting other organs. This paper discusses TRPM3’s expression in various organs and its potential impact on ME/CFS symptoms, with a focus on small nerve fibers and the brain, where TRPM3 is involved in presynaptic GABA release.

## Introduction

Myalgic Encephalomyelitis/Chronic Fatigue Syndrome (ME/CFS) is a common, debilitating disease often associated with conditions such as postural orthostatic tachycardia syndrome (POTS), orthostatic intolerance (OI), sympathetic hyperactivity, and small fiber neuropathy (SFN) [1–4]. ME/CFS shares many symptoms with Post-COVID Syndrome (PCS), and a subset of PCS patients develops the full spectrum of ME/CFS symptoms, increasing the prevalence of ME/CFS (designated as PCS-ME/CFS) [5, 6]. Despite the severity of symptoms and the identification of numerous cardiovascular, microvascular, and muscular abnormalities in scientific studies, no specific biomarker for ME/CFS has been identified. Recent studies have reported TRPM3 channel dysfunction in natural killer (NK) cells in ME/CFS patients, characterized by a decrease in calcium influx. This dysfunction has also been observed in PCS patients [7–16]. TRPM3 dysfunction is assessed as a decrease in the influx of calcium into the cells [16]. Dysfunctional TRPM3 channels in NK cells may contribute to ineffective pathogen clearance, potentially linking to post-infectious or immunological causes of ME/CFS and PCS. However, this connection is beyond the scope of this paper. Naltrexone has been shown to improve TRPM3 channel function in NK cells both in vitro and in vivo. Given its reported efficacy in low-dose treatment for ME/CFS, this finding prompts further investigation into TRPM3 channel disturbances in ME/CFS [9, 10, 16]. An isolated TRPM3 dysfunction in NK cells alone does not satisfactorily explain the pathophysiological role of TRPM3 in ME/CFS. Thus, the improvement in TRPM3 function by low-dose naltrexone (LDN) treatment does not fully account for its clinical efficacy. TRPM3 is also expressed in small nerve fibers and the brain. It is unlikely that ion channel dysfunction is limited to a single organ or cell type. TRPM3 dysfunction in small nerve fibers and the brain, affecting GABA release, could significantly contribute to ME/CFS pathophysiology. Improvements in TRPM3 function in these areas could better explain the effectiveness of LDN. This paper explores how TRPM3 dysfunction in small nerve fibers and the brain could be involved in ME/CFS pathophysiology, providing an indirect explanation for the efficacy of LDN, which has been shown to improve TRPM3 function in NK cells.

## Types of TRPM3 channel dysfunction

Mutations in human TRP channels can cause channelopathies, resulting in either gain-of-function or loss-of-function. These channelopathies lead to various diseases, depending on the affected TRP channel isoform, such as neurodevelopmental disorders, sensory deficits, and other systemic conditions [17]. In ME/CFS patients, investigations have revealed a loss-of-function mutation in TRPM3 channels in NK cells, evidenced by decreased calcium influx [16]. Conversely, two disease-associated variants of TRPM3 have been reported to cause a gain-of-function, resulting in cellular calcium overload due to increased basal TRPM3 activity [18]. These gain-of-function variants are associated with neurodevelopmental symptoms, including intellectual disability, epilepsy, musculoskeletal anomalies, and altered pain perception. Additionally, TRPM3 mutations have been linked to inherited cataract and glaucoma [19, 20]. In ME/CFS, the loss-of-function of TRPM3 is indicated by decreased calcium influx in NK cells, which can be ameliorated by low-dose naltrexone (LDN) treatment [16]. Currently, no other diseases are known to be associated with TRPM3 loss-of-function. Further research is needed to explore the broader implications of TRPM3 dysfunction in ME/CFS and its potential as a therapeutic target.

## Dysfunctional TRPM3 channels in patients suffering from ME/CFS

TRPM3 channels are widely expressed in both neuronal and non-neuronal tissues, including the brain, spinal cord, retina, pituitary, kidney, ovary, sensory nerves, vascular smooth muscle, skin, and testis [21–24]. TRPM3 channels play crucial roles in pain transduction, thermosensation, mechanoregulation, and vasorelaxation [25]. The hTRPM3 gene consists of 24 exons located on chromosome 9q-21.12, encoding a protein with 1555 amino acids and the characteristic six-transmembrane domain of the TRP family. Alternative splicing of the TRPM3 gene produces multiple channel isoforms with different cation permeabilities [21, 22, 26]. This alternative splicing can alter the primary sequence of the channel’s pore region, resulting in TRPM3 channels with distinct cation permeabilities, a unique feature among ion channels. We will now explore the potential consequences of TRPM3 dysfunction in small nerve fibers and the brain, highlighting their physiological roles in these organs. TRPM3 functions as a thermosensitive nociceptor channel, crucial for detecting noxious heat [27–29]. ME/CFS patients often exhibit dysregulated thermoregulatory responses and generalized pain without overt tissue damage, suggesting potential CNS impairments [10]. Dysfunction of TRPM3 channels could be implicated, as TRPM3 currents in NK cells from ME/CFS patients were resistant to ononetin (an antagonist) in the presence of pregnenolone and nifedipine (both agonists) [10]. Naltrexone, an antagonist to the µ-opioid receptor, can counteract the inhibitory effects of the µ-opioid receptor on TRPM3, thereby restoring TRPM3 ion channel activity [10]. Clinical studies indicate impaired TRPM3 channel activity in patients with post-COVID-19 conditions, suggesting that this dysfunction may contribute to chronic post-infection symptoms, similar to those observed in ME/CFS [15]. Symptoms induced by COVID-19, such as cough, smell and taste disturbances, loss of appetite, nausea, vomiting, inflammatory responses, and pain, can be linked to the dysfunction of TRP ion channels [29]. Interestingly, off-label use of LDN in clinical studies has shown alleviation of COVID-19 symptoms, including fibromyalgia, fatigue, and pain [30–33].

## Presence and role of TRPM3 in sensory nerves

Small nerve fiber degeneration has been consistently observed in some patients with ME/CFS and PCS [34–37]. Activation of TRPM3 has been shown to evoke the release of calcitonin gene-related peptide (CGRP) from sensory nerve terminals and perivascular nerve endings [38, 39]. Along with TRPV1, TRPM3 functions as a warm sensor and is involved in the secretion of neuropeptides such as Substance P, neurokinin A, and CGRP from sensory nerves [29, 38]. Functionally, these neuropeptides act as potent vasodilators. A deficit in neuropeptide secretion from sensory nerves may contribute to the observed imbalance favoring vasoconstriction over vasodilation in ME/CFS. CGRP plays a critical role in skeletal muscle function. Along with ß2-adrenergic receptors, CGRP stimulates Na^+^/K^+^-ATPase activity, which is essential for ionic homeostasis, excitability, and ion transport during exercise. Both receptors are the only stimuli for the Na^+^/K^+^-ATPase in skeletal muscle during exercise where the activity must rise by a factor of 10–20 over the level at rest. Insufficient Na^+^/K^+^-ATPase activity leads to sodium overload, which increases intracellular calcium levels. This can cause mitochondrial damage by reversing the sodium-calcium exchanger (NCX) to import calcium instead of exporting it. Finally, a deficit in CGRP has the potential to favor mitochondrial dysfunction. A recent study reported higher latency for warmth perception in ME/CFS and PCS patients, indicating disturbed warm sensation but normal cold perception [40]. TRPM3, along with TRPV1 and TRPA1, functions as a heat sensor. Heat sensing involves a trio of ion channels [41–43]. If structural damage or small nerve fiber degeneration were the cause, similar disturbances in cold sensing would be expected. The isolated disturbance of warmth sensing suggests a functional disturbance of the TRPM3 channel rather than degeneration [34–37]. So far, the isolated finding of a disturbance of warmth sensing rather speaks for a functional disturbance of the warmth sensor TRPM3 than for degeneration. While small nerve fiber degeneration is consistently reported in ME/CFS and PCS patients [34–37], it is plausible that functional TRPM3 defects in NK cells, potentially present early in the disease, contribute to this degeneration. It is difficult to believe that a functional defect of TRPM3 as found in natural killer cells and as it is perhaps present early on in the disease should not be finally involved in the degeneration of the small nerve fibers. TRPM3 dysfunction may contribute to nerve fiber degeneration, possibly in conjunction with other mechanisms such as microcirculatory disturbances, radicular compression, reactive oxygen species, and autoantibodies. Overall, TRPM3 dysfunction has significant pathophysiological potential, contributing to malperfusion, mitochondrial dysfunction, and small fiber degeneration in ME/CFS.

## TRPM3-expression and function in the brain

TRPM3 is ubiquitously expressed in multiple brain regions [44] and plays a crucial role in regulating the GABA system [45]. A deficit in TRPM3 can weaken the inhibitory GABA system. Pregnenolone sulphate (PS), a natural agonist, stimulates TRPM3 and has been used to identify TRPM3 deficits in NK cells. This section focuses on the direct effect of PS on the GABA system and how TRPM3 dysfunction can disrupt this effect. GABA has crucial inhibitory effects in the brain, counteracting the excitatory amino acid glutamate. PS stimulates GABA release presynaptically via TRPM3 but inhibits the GABA system postsynaptically through allosteric inhibition at the GABA receptor [45]. TRPM3 dysfunction impairs the stimulatory effect of PS on the GABA system, shifting the overall impact of PS towards inhibition. With a weakened GABA system due to TRPM3 dysfunction, the excitatory effects of glutamate remain unopposed. GABA is an important antagonist to the excitatory glutamate. Impairment of the dampening effect of the GABA system may allow the excitatory system e.g., glutamate to predominate. A recent magnetic resonance spectroscopy study found significantly elevated levels of glutamate and N-acetyl-aspartate in Long-COVID and ME/CFS patients compared to healthy controls, supporting the hypothesis of TRPM3 dysfunction contributing to excitatory imbalance [46]. Additionally, this may play a role in the hypervigilance and elevated sympathetic activity that are typical in ME/CFS and in skeletal muscle pathophysiology. Clinically, TRPM3 dysfunction may exacerbate stress levels in ME/CFS patients, who already experience high stress due to various factors, particularly orthostatic stress. The inability of PS to mitigate high stress levels may lead to brain overstimulation, increasing energy demands while substrate and oxygen delivery are compromised due to impaired perfusion [47, 48]. This energy mismatch can disrupt neurological function, leading to typical symptoms such as cognitive impairment, brain fog, and hypersensitivities to noise, light, and other sensory inputs [49]. TRPM3 dysfunction may also contribute to skeletal muscle pathophysiology, leading to muscle fatigue and post-exertional malaise (PEM), the hallmark of ME/CFS. The GABA system regulates muscle tone and tension, with GABA agonists like benzodiazepines and baclofen known to lower muscle tone and tension [50, 51]. Skeletal muscle pathophysiology is supposed to play a strong role in ME/CFS. Mental stress increases blood pressure, heart rate, and skeletal muscle tone, as assessed by EMG activity [52]. Increased muscle tone and activation in ME/CFS due to stress may elevate energy consumption and sodium influx, leading to intracellular sodium loading, disrupted calcium homeostasis, and ultimately, calcium overload and functional damage to skeletal muscle, contributing to PEM. TRPM3 dysfunction may exacerbate mental stress, indirectly impairing muscle perfusion via vasoconstriction and directly contributing to muscle pathophysiology and mitochondrial dysfunction. The mechanisms of sodium and calcium overload causing mitochondrial dysfunction have been extensively discussed previously [53]. Future research should further explore these connections to develop targeted therapies for ME/CFS. TRPM3-dysfunction may help to understand the enigmatic association of muscular pathology like muscle damage and skeleal muscle [54, 55], mitochondrial dysfunction and neurological symptom like hypervigilance in ME/CFS.

## Types of TRPM3 channel dysfunction

To determine the type of TRPM3 dysfunction present in NK cells of ME/CFS patients, investigations have shown a decreased influx of calcium, indicating a loss-of-function mutation [16]. Two disease-associated variants of TRPM3 lead to a gain-of-function, characterized by increased basal TRPM3 activity and resulting in cellular calcium overload [18]. Patients with these gain-of-function variants exhibit a broad spectrum of neurodevelopmental symptoms, including intellectual disability, epilepsy, musculoskeletal anomalies, and altered pain perception. Additionally, mutations in TRPM3 have been associated with inherited cataract and glaucoma [19]. In ME/CFS, there is clear evidence of a loss-of-function mutation in TRPM3, as indicated by decreased calcium influx, which can be improved by low-dose naltrexone (LDN) treatment [16]. Currently, no other diseases have been identified with a loss-of-function mutation in TRPM3.

## Conclusion

Investigating TRPM3 dysfunction in ME/CFS and PCS is crucial for advancing our understanding of the pathophysiology of these conditions. TRPM3 dysfunction is relevant to the pathophysiology of ME/CFS at three critical levels: in NK cells, sensory nerve fibers, and the brain.This dysfunction may contribute to the immunological disturbances, skeletal muscle dysfunction, and neurological symptoms observed in ME/CFS and PCS. Thus, TRPM3 dysfunction holds significant pathophysiological potential in relation to ME/CFS. Given the existence of multiple splice variants of the TRPM3 channel, each affecting channel properties significantly, it is important to investigate whether specific splice variants contribute to TRPM3 dysfunction and constitute significant risk factors for the development of ME/CFS. Improving TRPM3 dysfunction in small nerve fibers and the brain with low-dose naltrexone (LDN) may better explain its clinical efficacy in ME/CFS than solely targeting TRPM3 function in NK cells.

## Data Availability

Not applicable.
